# The Effects of Physical Activity on Cancer Patients Undergoing Treatment with Immune Checkpoint Inhibitors: A Scoping Review

**DOI:** 10.3390/cancers13246364

**Published:** 2021-12-18

**Authors:** Amy L. Shaver, Swapnil Sharma, Nikita Nikita, Daniel S. Lefler, Atrayee Basu-Mallick, Jennifer M. Johnson, Meghan Butryn, Grace Lu-Yao

**Affiliations:** 1 Department of Medical Oncology, Sidney Kimmel Cancer Center at Jefferson, Sidney Kimmel Medical College, Philadelphia, PA 19107, USA; Swapnil.Sharma@Jefferson.edu (S.S.); Fnu.Nikita@Jefferson.edu (N.N.); Daniel.Lefler@Jefferson.edu (D.S.L.); Atrayee.BasuMallick@Jefferson.edu (A.B.-M.); Jennifer.M.Johnson@Jefferson.edu (J.M.J.); Grace.LuYao@Jefferson.edu (G.L.-Y.); 2 Department of Psychology, Drexel University, Philadelphia, PA 19104, USA; mlb34@drexel.edu; 3 Jefferson College of Population Health, Philadelphia, PA 19107, USA

**Keywords:** immune checkpoint inhibitors, physical activity, exercise, exercise therapy, adverse events, tumor growth, concurrent therapy

## Abstract

**Simple Summary:**

Cancer treatments can cause adverse effects such as cancer-related fatigue. Immune checkpoint inhibitors (ICIs) are a relatively new therapy for some cancers and have shown great promise in helping people. Physical activity has been shown to aid many cancer patients to overcome adverse effects in traditional chemotherapy, but along with ICIs, it hasn’t been fully examined. This study was carried out to describe where the current research is now and to find knowledge gaps to help shape future research with ICIs, physical activity, and cancer outcomes.

**Abstract:**

Background: Cancer therapies are associated with multiple adverse effects, including (but not limited to) cancer-related fatigue (CRF). Fatigue is one of the most common side effects of immune checkpoint inhibitors (ICIs), occurring in up to 25% of patients. Physical activity has been shown to help reduce CRF through modulating the immune system, and may synergistically aid in the anti-tumor effects of ICIs. This review describes the nature and scope of evidence for the effects associated with concurrent physical activity while undergoing ICI therapy. Method: Scoping review methodology was utilized to identify studies, extract data, and collate and summarize results. Results: In literature published from January 2010 through to August 2021, only one human study and three pre-clinical studies met inclusion criteria. Conclusion: Existing evidence supports that physical activity is associated with decreased treatment-related toxicities such as CRF. However, further investigation is warranted. The dearth of clinical studies illustrates the need for more research to address this question, to guide patients and their providers in the application of appropriate physical activity interventions in those patients undergoing ICI.

## 1. Introduction

Immune checkpoint inhibitors (ICIs) have demonstrated clinical efficacy in multiple cancer settings. Since the initial Food and Drug Administration (FDA) approval for an ICI (ipilimumab) in 2011 for advanced stage melanoma, efficacy has been demonstrated in a broad range of both solid tumors and hematologic malignancies. ICIs are now indicated in the neoadjuvant, adjuvant, advanced/metastatic, and recurrent settings for various tumor types [1]. Additionally, pembrolizumab became the first anti-neoplastic medication approved across solid tumors solely based on a biomarker, as a result of early studies including the phase 2 KEYNOTE-158 trial [2]. As the indications for ICIs expand, so too must the medical community’s understanding of their effects, both on tumor biology and patient-reported outcomes.

While the use of ICIs has improved outcomes in cancer, ICIs are also associated with multiple adverse effects, such as cancer-related fatigue (CRF), which occurs in up to 25% of patients. Physical activity has been previously shown to be effective in the milieu of chemotherapy for decreasing the severity of chemotherapy- and cancer-related side effects [3,4,5]. Similarly, physical activity has been shown to reduce CRF and modulate the immune system through multiple mechanisms in cancer patients [6,7].

Therefore, it has been postulated that physical activity may impact the outcomes of those being treated with ICIs. Current recommendations are to be as physically active as an individual’s abilities will allow [8]. Cancer patients are recommended to follow guidelines for healthy populations, which may not always be appropriate [9], even though many cancer survivors have reportedly reaped great benefits from individualized fitness regimens [10].

The utilization of ICIs has increased exponentially, and along with this use there have been immune-related adverse events. Previous work indicates that physical activity concurrent with cancer therapy may be useful for alleviating adverse events in cancer patients; however, this is as yet unknown. The purpose of this scoping review is to describe how physical activity is conceptualized as concurrent cancer therapy to ICIs, to identify the evidence available in the field for the addition of physical activity to ICIs, to elucidate the outcomes of adding physical activity to ICIs, and to identify and analyze knowledge gaps in the field in order to further future research.

## 2. Materials and Methods

The scoping review was conducted according to the Joanna Briggs Institute methodology for scoping reviews, which is based predominantly on the protocols established by Arksey and O’Malley, but also includes the revisions suggested by Levac et al. and Peters et al. [11,12,13,14,15]. The review followed six steps: (1) defining the research question; (2) identifying relevant studies; (3) study selection; (4) charting the data; (5) collating, summarizing, and reporting the results; and (6) consultation. Reporting of findings was conducted according to Preferred Reporting Items for Systematic Reviews and Meta-Analyses (PRISMA) guidelines using the PRISMA extension for scoping reviews (PRISMA-ScR) checklist and is shown in Appendix A [16].

### 2.1. Inclusion and Exclusion Criteria

The scoping review was intended to include studies of individuals being treated with ICIs for those cancers for which the FDA has approved the use of ICIs as treatment as of 31 December 2020. The focus of the review was to clarify the *concept* of physical activity as concurrent therapy to ICIs, and to describe the outcomes of physical activity as concurrent therapy to ICIs in our participants of interest. Given the possibility of a scarcity of data, the decision was made to not limit the study to human studies. For *context*, the review included institutional and community care settings. No restrictions were placed on type of physical activity, so long as the activity was concurrent with administration of ICIs and the outcomes of both physical activity and concurrent ICI administration were reported. All full-text, peer-reviewed publications published from January 2010 through to August 2021 were included for consideration. January 2010 was chosen as a start date so as to capture any published trial data relating to the first ICI approval in 2011.

The search was restricted to articles and reports published in English. It was restricted to full-text, peer-reviewed articles so as to be certain of the scientific rigor of the results presented. Abstract-only publications were excluded, as were any papers that failed to fully elucidate outcomes (editorials, etc.). Though abstracts and editorials can be useful to help form search strategies, they can lack less-than-favorable results. By their abbreviated nature, abstracts do not present all data, and so were excluded. Editorials are opinion-only.

### 2.2. Search Strategy

The search strategy for this review was the result of prior research in the fields of prostate cancer and immunotherapy, as well as the strategies recommended by Tawfik et al. for adapting searches according to database [17]. An experienced search librarian was also consulted. The full protocol was registered at both Open Science Framework and Figshare (https://osf.io/kb8pq/?view_only=9df23d7dd1204049a05ff37b893874c8, accessed on 30 August 2021 and https://doi.org/10.6084/m9.figshare.16540152.v3, accessed on 30 August 2021 respectively).

The following databases were searched: PubMed, CINAHL, Cochrane Library, and Scopus. The search terms included (but were not limited to): “neoplasms”, “cancer”, “exercise”, “activity”, “physical activit*”, and “immune checkpoint inhibitor”. Further, MeSH terms and subject headings were also employed. A full search strategy was developed for PubMed and is shown in Appendix B.

### 2.3. Data Charting Process and Extraction Items

Three members of the research team (A.L.S., S.S., and N.N.) participated in the data extraction process. First, titles were screened for duplicates via software (EndnoteX9). Manual screening was conducted by the same three team members. Second, titles were screened manually for duplicates. Finally, titles and abstracts were screened based on inclusion/exclusion criteria. Screening was conducted independently, and the inclusion forms were compared for consistency. Upon agreement, full-text review was then conducted independently. From each article, the following information was charted: author, year of publication, title, ICI, country of origin, study type, study population, operationalization of exercise/physical activity, primary objective(s), and outcome(s)/summary. Blank inclusion and extraction forms are included in the Supplemental Materials.

### 2.4. Synthesis of Results

In the event that studies utilized similar physical activity and ICIs, a systematic review and meta-analysis would be the ideal synthesis tool. However, a narrative synthesis was instead chosen, given the broad scope of the question, the desire of the research team to allow for multiple forms of physical activity, and the relatively new nature of the field. The articles were grouped according to subject type (pre-clinical, clinical). As there was only 1 human study which included prospectively collected data and 3 murine randomized control trials, a critical appraisal of evidence within trials was deemed unnecessary for this review.

## 3. Results

### 3.1. Selection of Sources of Evidence

**Figure 1:**
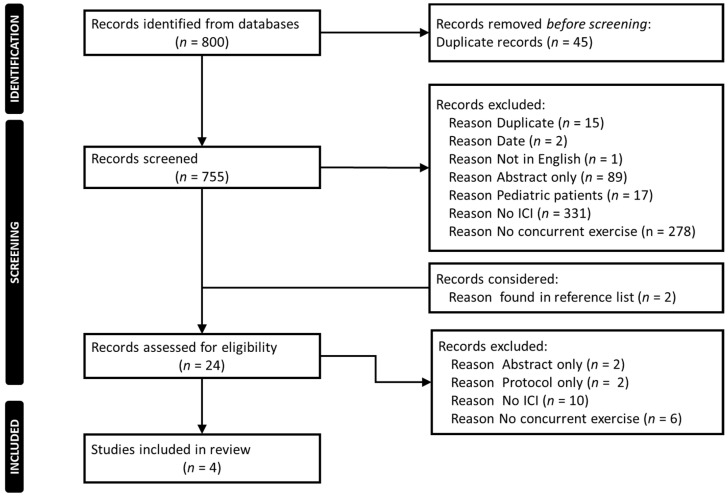
PRISMA Flow Chart of Studies included in the review.

The literature search retrieved 800 articles. Computer software eliminated 45 duplicates. An additional 15 duplicates were manually eliminated. The remaining 740 abstracts were screened and, with the addition of 2 articles found through other sources, 24 articles were identified for full-text review. After the full-text review, 4 articles remained for inclusion in the scoping review, having fulfilled all inclusion criteria. The most frequently cited reasons for article exclusion were lack of an ICI and lack of a concurrent physical activity intervention during ICI therapy (Figure 1).

### 3.2. Characteristics of Sources of Evidence

The studies’ characteristics are summarized in Table 1. The studies ranged in time of publication from 2018 to 2021. Three of the studies were pre-clinical randomized control trials [18,19,20] and one was a prospective clinical cohort pilot study [21]. The most common form of physical activity was running a treadmill [19,20], and PD-1/PD-L1 inhibitors were used in all studies. Two of the studies examined the response in melanoma tumors [18,21], one considered breast tumor response [19], and one utilized non-small cell lung cancer (NSCLC) [20].

### 3.3. Key Findings

The main outcomes measured in the clinical study were feasibility, patient-reported symptoms, anxiety and depression, toxicity, and patient adherence [21]. Multiple myeloma patients treated with pembrolizumab were recruited to test the feasibility of a multimodal support program. The program included care provided by physicians, dieticians, and exercise physiologists. The patients had low numbers of adverse events both pre- and post-study, the most prevalent being fatigue and sleep issues. The physical activity intervention was shown to be feasible with an 85% completion rate. Limitations of this study included the small sample size, the mixing of cohorts in which patients were both initiating and pre-established on treatment with ICIs, and the self-selective nature of the control group.

The main focuses of the pre-clinical studies were tumor size and growth rate [18,19,20]. All three murine studies indicated that physical activity slowed tumor growth, slowed immune cell proliferation, and improved immune sensitivity [18,19,20].

Bay et al. tested the addition of physical activity via voluntary wheel running to immune checkpoint blockade (either PD-1 or PD-L1) on tumor growth and gene expression of immune regulatory molecules [18]. Wheel running alone was found to increase expression of PD-1, PD-L1 and PD-L2 significantly. In this same model, tumor growth was reduced by 72% in mice who demonstrated voluntary physical activity when compared to the inactive control mice (*p* = 0.13). Mice who participated in voluntary physical activity and were treated with a PD-L1 inhibitor showed an 83% reduction in the rate of tumor growth (*p* < 0.05) compared to the rate of growth in the sedentary group. A 50% rate of tumor growth reduction was seen in mice with physical activity combined with PD-1 blockade (*p* = 0.07) compared to sedentary mice. The researchers found no additional advantage to administering both PD-1 and PD-L1 blockade along with physical activity.

Gomes-Santos et al. studied the effect of physical activity, concurrent with anti-PD-1 alone or in combination with anti-CTLA-4 treatment, on tumor growth and the tumor environment [19]. The study used mice to model human breast cancer and treadmills for physical activity. Physical activity was able to decrease the rate of tumor growth, as well as induce vessel normalization. Seven days of exercise training in three different models of breast cancer resulted in reduced tumor burden (approximately 30% decrease in tumor weight; model 1: ~600 mg vs. 400 mg; model 2: ~600 mg vs. 450 mg; model 3: ~825 mg vs. 600 mg). The tumor microenvironment of treadmill mice had increased perfusion and decreased hypoxia. There was no change in blood vessel density, but a positive impact was reported on the fraction of blood vessels that were perfused. RNA sequencing also confirmed that there was reprogramming of the tumor microenvironment towards more oxidative phosphorylation and less immunosuppression. These changes had important implications on the sensitivity of the breast cancer model; mice treated with a combination of immunotherapies directed at both PD-1 and CTLA-4 as well as scripted exercise had delayed tumor growth and decreased tumor volume compared to sedentary mice. Finally, physical activity prevented CRF in immune-checkpoint-blockade-treated mice, as measured by preservation of exercise capacity, demonstrated through increased time to exhaustion (~1500 sec vs. ~2500 sec; *p* < 0.001) and total running distance ~450 m vs. ~1000 m; *p* < 0.001) compared to the control group.

Martín-Ruiz utilized a murine model of non-small cell lung cancer (NSCLC) to study the effects of physical activity in combination with nivolumab therapy [20]. The animals in this study experienced both treadmill running to target aerobic capacity, as well as cage climbing and bar hanging for strength training. Similar to the Bay et al. study, physical activity alone reduced tumor growth rate in comparison to sedentary mice (*p* = 0.05). When nivolumab was added to the regimen, tumor death (*p* = 0.026) and apoptosis (*p* = 0.030) were increased among the physically active mice. As expected, aerobic capacity and strength improved in the active mice. Of note, neutrophil tumor infiltration was higher in the physically active group in combination with nivolumab group (*p* = 0.018) compared to the inactive group, and VEGF-A expression was higher in the nivolumab group, regardless of physical activity status.

## 4. Discussion

The aim of this scoping review was to elucidate the extent of published research evaluating the effects of concurrent physical activity interventions and the use of ICIs. To the authors’ knowledge, this is the first scoping review to focus on studies that assess the concurrent administration of ICIs and a physical activity regimen. Our results identify a major gap in human-based research in the field. Part of the reason for this may be the relatively new nature of the medications that are still undergoing trials for approvals in different cancer types. Another reason may be a lack of trained practitioners to aid patients as described by Santa Mina et al. [22] This may have led to human studies being proposed but not reaching completion, and therefore remaining unpublished. Many of the studies published to date have been during the survivorship phase rather than during active treatment, which limits the availability of data on the direct synergistic effect of physical activity on cancer therapy.

As a concept, physical activity is considered to be complementary to immune checkpoint blockade. From pre-clinical to clinical studies, “physical activity” was viewed as anything from wheel-running, to strength training, to walking, to qi-gong and yoga. The pre-clinical studies indicate that the combination of physical activity and checkpoint inhibition is advantageous to the patient through decreased tumor growth, improved strength, decreased fatigue, and improved ability of the immune system to fight cancer.

### 4.1. Physiology of Exercise and Immunology

There is a growing body of evidence investigating the mechanisms by which exercise modulates immunity. Much of this research points to effects on natural killer (NK) and T cells, rather than to components of humoral immune responses [7]. These are also the immune cells that are targeted by ICIs, indicating that the anti-tumor effects of both exercise and checkpoint blockade may be synergistic.

It has been shown that sedentary patients have higher proportions of both CD4+ and CD8+ T cells that express PD-1, a negative immunologic regulator [23]. Meanwhile, CD8+ cytotoxic T cells are mobilized by acute exercise, and thus more able to participate in active immunity [24]. Exercise further induces the proliferation and activation of T cells against tumors, likely through adrenergic stimulation [25]. Finally, T cells that undergo repeated stimulation suffer from both senescence (a decreased ability to replicate partially due to telomere shortening) and exhaustion (the loss of vital functions). However, these two processes of immune impairment are attenuated by the effects of exercise [26].

As the blockade of PD-L1, PD-1, and CTLA-4 by ICIs results in the activation of T cells, it stands to reason that this effect would be augmented by exercise through the above mechanisms. In fact, this was demonstrated in a mouse model of breast cancer, which showed that exercise slowed immunosuppressive elements of the tumor microenvironment and induced increases in CD8+ T cell activation [27]. This was tested in the presence of radiotherapy (RT) plus PD-1 blockade. The investigators found that the addition of exercise to RT+PD-1 blockade increased splenic CD8+ T cells, decreased PD-1 expression on NK cells, increased markers of NK-cell activation, and ultimately slowed tumor growth.

Although there are clearly physiologic reasons for synergism with immunotherapies, physical activity has also been shown to improve outcomes when combined with chemotherapy. One study in breast cancer patients found that a physical activity regimen was adhered to more closely while patients were undergoing therapy, as compared with when after therapy was complete; and higher adherence occurred during chemotherapy than during radiotherapy [28]. In lung cancer, across the cancer continuum, increased physical activity was found to be safe and sought-after by patients, and shown to improve quality of life [29].

Similarly, a recent study indicated that the combination of diet, physical activity, and chemotherapy improved the efficacy of chemotherapy in patients with acute lymphoblastic leukemia [30]. Compared to usual care, those patients who participated with a diet and physical activity program during treatment saw a reduction in minimal residual disease.

Similarly to the murine results of the Martín-Ruiz et al. study, Reis et al. found an increase in in both functional and aerobic capacity in human breast cancer patients undergoing treatment [20,31]. Reis et al. also found a decrease in pain scores and an increase in strength for those undergoing a physical activity regimen during their chemotherapy. There was no significant finding for fatigue. Likewise, the OptiTrain group found lower rates of thrombocytopenia in their exercising group compared to usual care [32].

### 4.2. Strengths and Limitations

This scoping review had a number of strengths. The review used a strong and transparent methodology. A protocol was followed and was registered before research began. A broad search of the literature was conducted in four databases. Finally, the review was conducted by a multidisciplinary team. The review also had some limitations. To manage scope, we excluded studies without clearly defined outcomes, as well as abstracts. However, the abstracts that were eliminated on full scan could have also been eliminated for lack of concurrent therapy. Another limitation is the use of articles published only in English in the review. Articles examining the topic in other languages may have been missed in the search. The study included only four articles, and so the synthesis of results is limited.

## 5. Conclusions

The results of the scoping review suggest that the current availability of research is lacking to inform the use of concurrent administration of physical activity or increased physical activity and ICIs. Pre-clinical studies suggest that the addition of physical activity, whether as a prescribed regimen or as a voluntary practice, has benefits both in tumor growth rate and volume. Those studies also show an improvement in strength and in immune response. The clinical pilot study showed efficacy for the addition of physical activity to immunotherapy. Prior studies indicate that the addition of physical activity benefits chemotherapy. There is a need now to perform more clinical studies combining physical activity with immunotherapy, so as to inform clinicians and improve outcomes for patients.

## Figures and Tables

**Table 1:** Selected study characteristics.

	Authors	Study Design	Population Characteristics	ICI	Physical Activity	Outcome
Clinical/Human						
1	Lacey, J. et al. (2018) [21]	Pre-/Post-test cohort design	N = 28 MM patients; 13 intervention, 15 control; (3 non-complete); age 42–85, median 66; 16 male, 12 female; median 2.75 years since diagnosis	Pembrolizumab	1 h consultation w/exercise physiologist to design an exercise program that included 16 sessions of physical activity tailored to patient’s preferences and capabilities and an activity monitor; review throughout 9-week trial and follow-up at completion of 9 weeks; included aerobic, resistance, and other (qi gong, yoga, etc.)	Adherence, patient-reported symptoms, anxiety and depression, and toxicity
Pre-Clinical/Murine						
2	Bay, M.L. et al. (2020) [18]	RCT	8–16 weeks old C57BL/6 mice with subcutaneous tumors (B16 melanoma tumors); all female; 4 groups N = 14 (control sedentary, control exercising, treated sedentary, and treated exercising); identical studies of PD-L1 and PD-1 inhibitors	PD-L1 and PD-1 inhibitor treatment After tumor inoculation, injections were 3x per week for 2 weeks	Voluntary wheel running Mice had access to wheels for 5 weeks prior to study	Immune response in an immunologically ‘cold’ tumor; Tumor growth, changes in body weight and spleen weight
3	Gomes-Santos, I.L. et al. (2021) [19]	RCT	8–10 weeks old female C57BL/6, FVB, Balb/c mice; breast tumor tissue at 100 mm^3^ signaled study start; CD8^+^T cells depleted prior to study start; *n* = 6 mice per group	Immune checkpoint blockade (ICB): anti-PD-1 alone, anti-PD-1 with anti-CTLA-4 or IgG administered concurrent with ExTr	Treadmill to mimic moderate-to-vigorous intensity prescribed by American College of Sports Medicine 30–60 min. 3–5 d/wk; exercise training of 45 min/d treadmill time at 60% maximal velocity	Time for tumor growth; tumor and surrounding vasculature; immune cell counts
4	Martín-Ruiz, A. et al. (2020) [20]	RCT	Human NSCLC tissue (previously untreated basaloid infiltrating squamous cell stage IIA) and patient derived xenograft (PDX) mice; 8-week-old female mice; 100 mm^3^ tumor size included; non-exercise control *n* = 5, exercise control *n* = 5, exercise + nivolumab *n* = 6, non-exercise + nivolumab *n* = 6	Nivolumab	Aerobic and resistance training 5 days per week; aerobic 5 days/week: treadmill work up to 80% max velocity, strength 2 days per week: horizontal screen exercise (climbing), hanging with two limbs; 8-week intervention	Aerobic capacity, forelimb grip strength, tumor volume and growth rate, cell proliferation, apoptosis,

## Data Availability

The data reported in this study can be obtained through writing to A.L.S., the corresponding author.

## Supplementary Materials

The following are available online at https://www.mdpi.com/article/10.3390/cancers13246364/s1, supplemental materials 1: Blank inclusion and extraction forms.

## Appendix A. Preferred Reporting Items for Systematic Reviews and Meta-Analyses Extension for Scoping Reviews (PRISMA-ScR) Checklist

**Table A1:** PRISMA-ScR Checklist.

Section	Item	PRISMA-ScR Checklist Item	Reported on Page
Title			
Title	1	Identify the report as a scoping review.	1
Abstract			
Structured summary	2	Provide a structured summary that includes (as applicable): background, objectives, eligibility criteria, sources of evidence, charting methods, results, and conclusions that relate to the review questions and objectives.	1
Introduction			
Rationale	3	Describe the rationale for the review in the context of what is already known. Explain why the review questions/objectives lend themselves to a scoping review approach.	2
Objectives	4	Provide an explicit statement of the questions and objectives being addressed with reference to their key elements (e.g., population or participants, concepts, and context) or other relevant key elements used to conceptualize the review questions and/or objectives.	2
Methods			
Protocol and registration	5	Indicate whether a review protocol exists; state if and where it can be accessed (e.g., a Web address); and if available, provide registration information, including the registration number.	2–3
Eligibility criteria	6	Specify characteristics of the sources of evidence used as eligibility criteria (e.g., years considered, language, and publication status), and provide a rationale.	2
Information sources	7	Describe all information sources in the search (e.g., databases with dates of coverage and contact with authors to identify additional sources), as well as the date the most recent search was executed.	2
Search	8	Present the full electronic search strategy for at least 1 database, including any limits used, such that it could be repeated.	Appendix Table A2
Selection of sources of evidence	9	State the process for selecting sources of evidence (i.e., screening and eligibility) included in the scoping review.	2
Data charting process	10	Describe the methods of charting data from the included sources of evidence (e.g., calibrated forms or forms that have been tested by the team before their use, and whether data charting was performed independently or in duplicate) and any processes for obtaining and confirming data from investigators.	3
Data items	11	List and define all variables for which data were sought and any assumptions and simplifications made.	3
Critical appraisal of individual sources of evidence	12	If carried out, provide a rationale for conducting a critical appraisal of included sources of evidence; describe the methods used and how this information was used in any data synthesis (if appropriate).	3
Synthesis of results	13	Describe the methods of handling and summarizing the data that were charted.	3
Results			
Selection of sources of evidence	14	Give numbers of sources of evidence screened, assessed for eligibility, and included in the review, with reasons for exclusions at each stage, ideally using a flow diagram.	4
Characteristics of sources of evidence	15	For each source of evidence, present characteristics for which data were charted and provide the citations.	4–6
Critical appraisal within sources of evidence	16	If carried out, present data on critical appraisal of included sources of evidence (see item 12).	3
Results of individual sources of evidence	17	For each included source of evidence, present the relevant data that were charted that relate to the review questions and objectives.	4–6
Synthesis of results	18	Summarize and/or present the charting results as they relate to the review questions and objectives.	4, 6
Discussion			
Summary of evidence	19	Summarize the main results (including an overview of concepts, themes, and types of evidence available), link to the review questions and objectives, and consider the relevance to key groups.	7
Limitations	20	Discuss the limitations of the scoping review process.	8
Conclusions	21	Provide a general interpretation of the results with respect to the review questions and objectives, as well as potential implications and/or next steps.	8
Funding	22	Describe sources of funding for the included sources of evidence, as well as sources of funding for the scoping review. Describe the role of the funders of the scoping review.	9

## Appendix B

**Table A2:** PubMed Search.

Database	Search Terms	Results
PubMed	(“bladder cancer”[All Fields] OR “bc”[All Fields] OR “cancer*”[All Fields] OR “cc”[All Fields] OR “cervical cancer”[All Fields] OR “cHL”[All Fields] OR “classical Hodgkin’s lymphoma”[All Fields] OR “Colorectal Neoplasms”[MeSH Terms] OR “metastatic colorectal cancer”[All Fields] OR “colorectal cancer”[All Fields] OR “CRC”[All Fields] OR “CSCC”[All Fields] OR “cutaneous squamous cell carcinoma”[All Fields] OR “endometrial cancer”[All Fields] OR “ec”[All Fields] OR “Endometrial Neoplasms”[MeSH Terms] OR “esophageal squamous cell carcinoma”[All Fields] OR “ESCC”[All Fields] OR “gastric carcinoma”[All Fields] OR “gc”[All Fields] OR “gastroesophageal junction carcinoma”[All Fields] OR “GEJ carcinoma”[All Fields] OR “head and neck cancer”[All Fields] OR “HNC”[All Fields] OR “HNSC”[All Fields] OR “Head and Neck Neoplasms”[MeSH Terms] OR “hepatocellular carcinoma”[All Fields] OR “HCC”[All Fields] OR “locally advanced”[All Fields] OR “lymphoma*”[All Fields] OR “Lymphoma”[MeSH Terms] OR “Melanoma”[MeSH Terms] OR “MCC”[All Fields] OR “Merkel cell carcinoma”[All Fields] OR “metastatic Merkel Cell carcinoma”[All Fields] OR “metastatic melanoma”[All Fields] OR “metastatic squamous NSCLC”[All Fields] OR “metastatic NSCLC”[All Fields] OR “metastatic non-squamous NSCLC”[All Fields] OR “non-squamous NSCLC”[All Fields] OR “carcinoma, non-small cell lung”[MeSH Terms] OR “carcinoma”[All Fields] OR “non-small cell”[All Fields] OR “lung”[All Fields] OR “non-small-cell lung carcinoma”[All Fields] OR “nsclc”[All Fields] OR “non-small cell lung cancer”[All Fields] OR “unresectable stage III NSCLC”[All Fields] OR “Neoplasms”[MeSH Terms] OR “neoplasia*”[All Fields] OR “pm”[All Fields] OR “pleural mesothelioma”[All Fields] OR “PMBCL”[All Fields] OR “primary mediastinal large B cell lymphoma”[All Fields] OR “advanced RCC”[All Fields] OR “RCC”[All Fields] OR “renal cell carcinoma”[All Fields] OR “small cell lung cancer”[All Fields] OR “SCLC”[All Fields] OR “solid tumor”[All Fields] OR “squamous cell head and neck cancer”[All Fields] OR “triple-negative breast cancer”[All Fields] OR “TNBC”[All Fields] OR “unresectability”[All Fields] OR “unresectable”[All Fields] OR “unresected”[All Fields] OR “urinary bladder neoplasms”[All Fields] OR “urothelial carcinoma”[All Fields] OR “metastatic urothelial carcinoma”[All Fields])	360
	AND (“exercise*”[All Fields] OR “Exercise”[MeSH Terms] OR “Exercise Therapy”[All Fields] OR “Exercise Therapy”[MeSH Terms] OR (“physical examination”[MeSH Terms] OR “physical examination”[All Fields] OR “physical”[All Fields] OR “physically”[All Fields] OR “physicals”[All Fields] OR “physical activit*”[All Fields] OR “weight-bearing exercise”[All Fields] OR “weight-bearing”[All Fields] OR “weight-bearing training”[All Fields] OR “strength training”[All Fields] OR “training”[All Fields] OR “aerobic”[All Fields] OR “aerobically”[All Fields] OR “Exercise”[All Fields] OR “aerobics”[All Fields] OR “aerobic training”[All Fields] OR “aerobic exercise”[All Fields] OR “aerobic activit*”[All Fields] OR “rehabilitant”[All Fields] OR “rehabilitants”[All Fields] OR “rehabilitate”[All Fields] OR “rehabilitated”[All Fields] OR “rehabilitates”[All Fields] OR “rehabilitating”[All Fields] OR “Rehabilitation”[MeSH Terms] OR “Rehabilitation”[All Fields] OR “rehabilitations”[All Fields] OR “rehabilitative”[All Fields] OR “Rehabilitation”[MeSH Subheading] OR “rehabilitations”[All Fields] OR “rehabilitational”[All Fields] OR “rehabilitator”[All Fields] OR “rehabilitators”[All Fields] OR “physical rehabilitation”[All Fields] OR “HIIT”[All Fields] OR “high intensity interval training”[All Fields] OR “therapeutic exercise”[All Fields] OR “aerobic conditioning”[All Fields] OR “rehabilitative exercise”[All Fields] OR “physical therapy modalities”[MeSH Terms] OR (“physical”[All Fields] AND “therapy”[All Fields] AND “modalities”[All Fields]) OR “physical therapy modalities”[All Fields] OR “physiotherapies”[All Fields] OR “physiotherapy”[All Fields] OR “resistance training”[All Fields] OR “Exercise Movement Techniques”[MeSH Terms]))	
	AND (“immunomodulatory”[All Fields] OR “immune therapy”[All Fields] OR “immune checkpoint inhibitors”[All Fields] OR “ICI”[All Fields] OR “anti-PD-1 monoclonal antibody”[All Fields] OR “pembrolizumab”[Supplementary Concept] OR “pembrolizumab”[All Fields] OR “pembrolizumab”[Supplementary Concept] OR “pembrolizumab”[All Fields] OR “keytruda”[All Fields] OR “anti-CTLA-4 monoclonal antibody”[All Fields] OR “tremelimumab”[Supplementary Concept] OR “tremelimumab”[All Fields] OR “nivolimumab”[All Fields] OR “nivolumab”[MeSH Terms] OR “nivolumab”[All Fields] OR “opdivo”[All Fields] OR “ipilimumab”[MeSH Terms] OR “ipilimumab”[All Fields] OR “ipilimumab”[MeSH Terms] OR “ipilimumab”[All Fields] OR “yervoy”[All Fields] OR “avelumab”[Supplementary Concept] OR “avelumab”[All Fields] OR “avelumab”[Supplementary Concept] OR “avelumab”[All Fields] OR “bavencio”[All Fields] OR “durvalumab”[Supplementary Concept] OR “durvalumab”[All Fields] OR “cemiplimab”[Supplementary Concept] OR “cemiplimab”[All Fields] OR “durvalumab”[Supplementary Concept] OR “durvalumab”[All Fields] OR “imfinzi”[All Fields] OR “cemiplimab”[Supplementary Concept] OR “cemiplimab”[All Fields] OR “libtayo”[All Fields] OR “atezolizumab”[Supplementary Concept] OR “atezolizumab”[All Fields] OR “tecentriq”[All Fields] OR “PD1”[All Fields] OR “PDL1”[All Fields] OR “CTLA4”[All Fields] OR “PD-1”[All Fields] OR “PD-L1”[All Fields] OR “ctla 4 antigen”[MeSH Terms] OR “ctla 4 antigen”[All Fields] OR “ctla 4”[All Fields])	
	AND (2010/01/01:2021/08/31[Date-Publication] AND “english”[Language]))) AND ((fft[Filter]) AND (2010/1/1:2021/8/31[pdat]) AND (english[Filter]) AND (alladult[Filter]))	
