# Supplementary material for: The Effects of Physical Activity on Cancer Patients Undergoing Treatment with Immune Checkpoint Inhibitors: A Scoping Review
Source: Cancers (Basel). 2021 Dec 18;13(24):6364. doi: 10.3390/cancers13246364 (PMC8699800; doi:10.3390/cancers13246364)
Supplement: Supplementary file 1 [file cancers-13-06364-s001.zip › cancers-1491423-supplementary.pdf]

Inclusion Assessment Form

Physical activity and immune checkpoint inhibitor (ICI) treatment

Reviewer: ____________________ Date: ____________

Reference: ______________________________________

____

Instructions: Complete the form on each study. If the final decision is “no”, exclude that study.

CRITERIA

	Yes	No	Unsure
1. Study Publication			
Is the study a full manuscript?	[ ]	[ ]	[ ]
Is the study an abstract only?	[ ]	[ ]	[ ]
Was the study published between January 2010 and August 2021?	[ ]	[ ]	[ ]
Was the study published in English?	[ ]	[ ]	[ ]
2. Study Population			
Did the study include patients aged 18 and over?	[ ]	[ ]	[ ]
Were patients receiving treatment with an ICI[#] for an FDA-approved condition[*]?	[ ]	[ ]	[ ]
3. Study Intervention			
Did the study include some form of physical activity/ exercise[^] concurrent with ICI therapy?	[ ]	[ ]	[ ]
4. Study Outcomes			
Did the study report on one or more outcomes?	[ ]	[ ]	[ ]

DECISION OF REVIEWER

	Yes	No	Unsure
1. Is this study potentially relevant for this review?	[ ]	[ ]	[ ]

CONSENSUS

Second review
- [ ] Include
- [ ] Exclude
- [ ] Disagree

Final consensus
- [ ] Include
- [ ] Exclude
- [ ] Disagree

# ICIs of interest

Keytruda (pembrolizumab)
Opdivo (nivolumab)
Libtayo (cemiplimab)
Tecentriq (atezolizumab)
Bavencio (avelumab)
Imfinzi (durvalumab)
Yervoy (ipilimumab)

***List of approved cancerous indications as of December 2020**

Pleural mesothelioma
Triple-negative breast cancer
Bacillus Calmette-Guérin bladder cancer
Cervical cancer
Classical Hodgkin's lymphoma
Colorectal cancer
Cutaneous squamous cell carcinoma
Endometrial carcinoma
Esophageal squamous cell carcinoma
Gastric cancer
Hepatocellular carcinoma
Head and neck squamous cell carcinoma
Merkel cell carcinoma
Non-small cell lung cancer
Primary mediastinal large B cell lymphoma
Renal cell carcinoma
Small cell lung cancer
Urothelial carcinoma

^ Consider a very broad definition of physical activity, including:

exercise OR "exercise therapy" OR "physical activity" OR "weight-bearing exercise" OR "weight-bearing" OR "weight-bearing training" OR "strength training" OR training OR aerobic OR "aerobic training" OR "aerobic exercise" OR "aerobic activity" OR rehabilitation OR "physical rehabilitation" OR "HIIT" OR "high intensity interval training" OR "therapeutic exercise" OR "aerobic conditioning" OR "rehabilitative exercise" OR physiotherapy OR "resistance training", etc

Extraction/Charting Form

Author(s)*	Country	Study type	Population	ICI	Exercise	Objective/Outcomes	Summary

* Include year, title, etc.
